# Metabarcoding reveals a high diversity and complex eukaryotic microalgal community in coastal waters of the northern Beibu Gulf, China

**DOI:** 10.3389/fmicb.2024.1403964

**Published:** 2024-06-05

**Authors:** Chaofan Wang, Junning Gu, Weiguo Li, Jian Wang, Zhaohui Wang, Qiuqi Lin

**Affiliations:** College of Life Science and Technology, Jinan University, Guangzhou, China

**Keywords:** microalgae, phytoplankton, Beibu Gulf, metabarcoding, microscopy, harmful algal blooms (HABs)

## Abstract

Beibu Gulf is an important semi-enclosed bay located in the northwestern South China Sea, and is famous for its high bio-productivity and rich bio-diversity. The fast development along the Beibu Gulf Economical Rim has brought pressure to the environment, and algal blooms occurred frequently in the gulf. In this study, surface water samples and micro-plankton samples (20–200 μm) were collected in the northern Beibu Gulf coast. Diversity and distribution of eukaryotic planktonic microalgae were analyzed by both metabarcoding and microscopic analyses. Metabarcoding revealed much higher diversity and species richness of microalgae than morphological observation, especially for dinoflagellates. Metabarcoding detected 144 microalgal genera in 8 phyla, while microscopy only detected 40 genera in 2 phyla. The two methods revealed different microalgal community structures. Dinoflagellates dominated in microalgal community based on metabarcoding due to their high copies of 18 s rRNA gene, and diatoms dominated under microscopy. Altogether 48 algal bloom and/or toxic species were detected in this study, 34 species by metabarcoding and 19 species by microscopy. Our result suggested a high potential risk of HABs in the Beibu Gulf. Microalgal community in the surface water samples demonstrated significantly higher OTU/species richness, alpha diversity, and abundance than those in the micro-plankton samples, although more HAB taxa were detected by microscopic observations in the micro-plankton samples. Furthermore, nano-sized taxa, such as those in chlorophytes, haptophytes, and chrysophyceans, occurred more abundantly in the surface water samples. This study provided a comprehensive morphological and molecular description of microalgal community in the northern Beibu Gulf.

## Introduction

Microalgae are the most important primary productors of the marine ecosystems, and play a key role in the global biogeochemical processes (Vogt, 2015; Worden et al., 2015). Microalgal community structure is regulated by a combination of physical, chemical, and biological factors, and fluctuates greatly to environmental variations (Raymont, 1983). Therefore, microalgal community structure can act as an indicator for environmental assessment of the marine ecosystems. However, some microalgal taxa form harmful algal blooms (HABs) and even produce toxins, which have negative impacts on marine organisms, cause serious economic losses to fisheries, aquaculture and tourism activities, as well as produce significant impacts on the environment and human health (Hallegraeff et al., 2004). About 177 marine microalgal species are listed as harmful microalgae, 117 of which belong to dinoflagellates (Lundholm et al., 2009). HABs occurred frequently in the worldwide coastal areas in the recent decades due to the global warming and eutrophication caused by the increase nutrient loading from human activities (Gilbert, 2017), especially in the Chinese coastal sea areas (Xiao et al., 2019; Sakamoto et al., 2021). Blooms caused by toxic flagellates especially those of dinoflagellates increased greatly in the China seas (Li et al., 2021; Tian et al., 2021; Wang et al., 2023).

**Figure 1:**
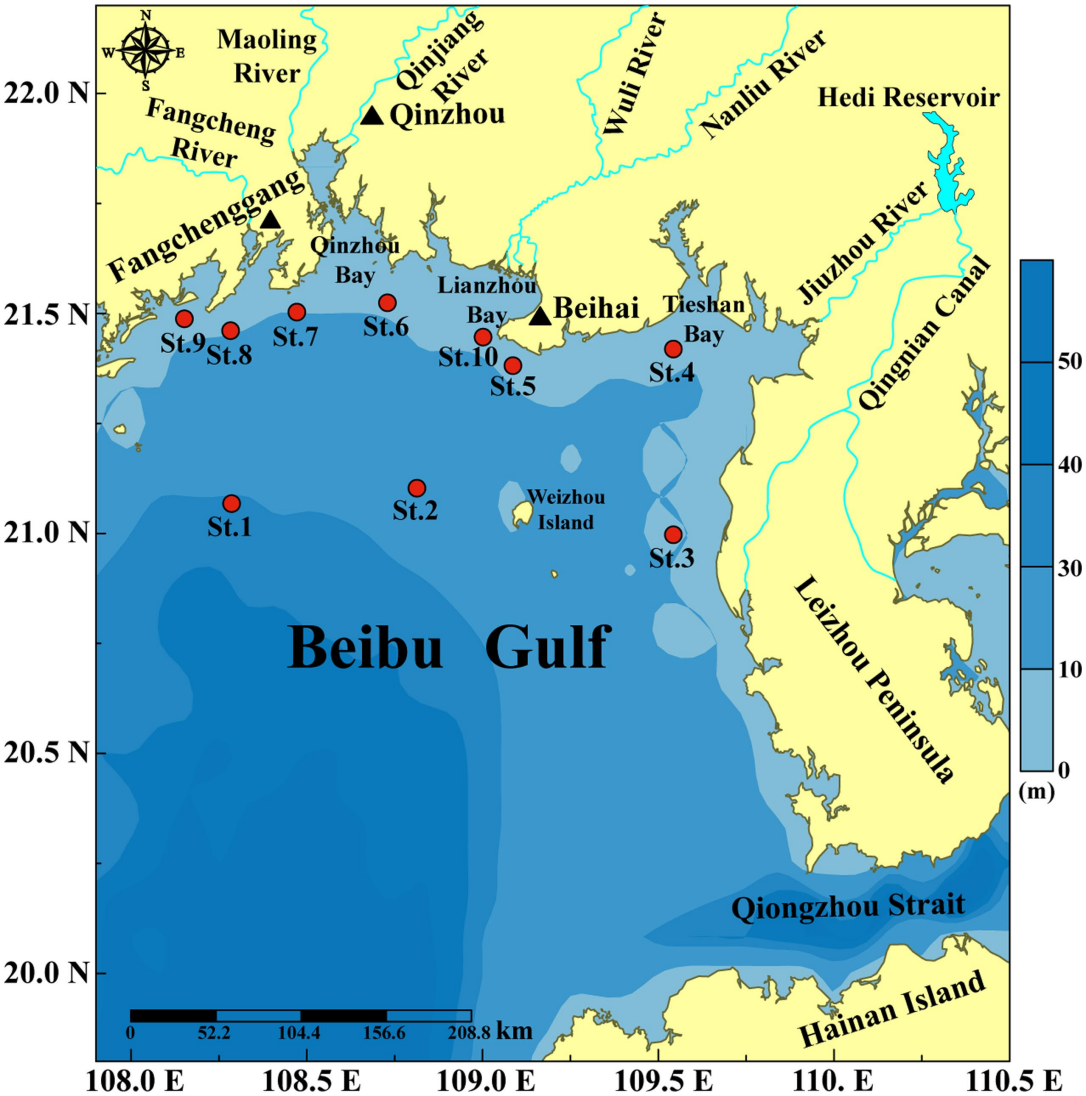
Sampling stations along the northern Beibu Gulf coast of China.

Beibu Gulf (16°00′–21°30′N, 105°40′–111°00′E) is an important semi-enclosed bay located in the northwestern South China Sea (SCS). It connects to the open SCS by the Qiongzhou Strait in the northeast and to the open sea in the south (Figure 1). The gulf is famous for its high bio-productivity and rich bio-diversity (Gan et al., 2013; Chen et al., 2018). Although water quality in the Beibu Gulf is generally good, the fast development along the Beibu Gulf Economical Rim has brought pressure to the environment (Kaiser et al., 2013; Lai et al., 2014; Lao et al., 2019). Some coastal sea areas, such as Qinzhou Bay, Fangchenggang Bay and Lianzhou Bay, have been undertaking severe environmental problems, such as eutrophication (Lai et al., 2014; Lao et al., 2021; Pan et al., 2021) and HABs (Xu et al., 2019; Wang et al., 2021; Niu et al., 2022; Xie et al., 2023). Before 2000, the cyanobacterium *Microcystis aeruginosa* was the dominant bloom species in the Beibu Gulf, and blooms of cyanobacteria, dinoflagellates, and diatoms co-occurred during the period 2001–2010, and the haptophyte *Phaeocystis globosa* has become a major causative species of HABs after 2011 (Xu et al., 2019; Wang et al., 2021; Gibson et al., 2022; Zhu et al., 2023). A massive bloom caused by the dinoflagellate *Noctiluca scintillans* with total coverage area over 20,000 km^2^ occurred in February 2021 in Beibu Gulf (Xie et al., 2023). Frequent HABs have pressed adverse impacts on local ecosystems, and Beibu Gulf has become a hot area for HAB study in China (Xu et al., 2019; Wang et al., 2021; Gibson et al., 2022; Xie et al., 2023; Zhu et al., 2023).

Many previous studies have reported microalgal community structure in Beibu Gulf based on microscopic analysis (Xu et al., 2014; Wang et al., 2015; Qin et al., 2023), pigment analysis combined with high performance liquid chromatography (HPLC)-CHEMTAX analysis (Wang et al., 2021; Pan et al., 2023), flow cytometry (Wang J. X. et al., 2022; Xu et al., 2022), and metabarcoding during the *Phaeocystis* blooms (Gibson et al., 2022; Zhu et al., 2023). However, most of these studies are based on one method alone, which cannot fully reflect microalgal diversity and distribution of HAB taxa.

A large proportion of HAB taxa belong to the micro-sized microalgae (20–200 μm), for example, most of the harmful dinoflagellates and all of the harmful raphidophyceans (Hallegraeff et al., 2004). Although diatom cells are usually <20 μm, most of the harmful and/or bloom diatoms can form chains with the length > 20 μm such as *Chaetoceros* spp., *Pseudo-nitzschia* spp., *Skeletonema* spp., *Thalassiosira* spp., and *Thalassionema* spp. (Hallegraeff et al., 2004). Therefore, a plankton net with mesh size of 20 μm is suggested to qualitatively concentrate microalgae especially those of HAB taxa (Anderson and Throndsen, 2004). In this study, surface water samples and micro-plankton samples (20–200 μm) were collected in the northern Beibu Gulf coast. Metabarcoding of the V4 region of 18S rRNA gene was performed to investigate diversity and distribution of eukaryotic microalgae with the focus on HAB species, and the results were compared with those based on microscopic observation. The purpose of this study is to assess microalgal diversity in the northern Beibu Bay by the combination of microscopic and metabarcoding analyses, and to understand the distribution of HAB species and the potential risks of HABs.

## Materials and methods

### Studying area and sample collection

The northern Beibu Gulf covers an area of 1.3 × 10^5^ km^2^, with an average water depth of 38 m and a 1,629 km coastline (Chen et al., 2011; Li et al., 2014). The average annual water temperature is *ca.* 24.5°C. The warm weather together with abundant nutrient inputs from coastal rivers, such as the Nanliu and Qinjiang rivers, makes this region one of the most abundant fishing grounds in China (Chen et al., 2011).

Samples were collected during the autumn survey supported by Guangxi Zhuang Autonomous Region Marine Environment Monitoring Center in November 2020. Surface water samples were collected at 10 stations (Figure 1) from a depth of 2.0 m by a submerged pump. Surface water was filtered through a 200 μm mesh to remove large zooplankton. One liter filtered water sample were fixed in 1.5% Lugol’s iodine solution, concentrated to a final volume of 20 mL before microscopic observation. Triplicate 1.0 L filtered water samples were filtered by 0.45 μm polycarbonate membrane (Millipore, United States) for metabarcoding analysis, and stored at −80°C until DNA extraction. And the filtrate was used for nutrient analysis.

Microplankton samples were collected at a depth of 2 m using a double-layered net with mesh sizes between 20 and 200 μm. A known volume of seawater (20 L) was pumped through the double-layered net and concentrated to 500 mL, 100 mL of which was fixed with 1.5% Lugol’s iodine solution for microscopic observation after concentration to a final volume of 20 mL, and the other three 100 mL samples were filtered through 0.45 μm polycarbonate membranes for metabarcoding analysis.

### Analysis of environmental parameters

Water temperature, salinity, pH, dissolved oxygen (DO), and chlorophyll *a* (Chl *a*) were measured by *in situ* a Multiparameter Sonde YSI 6600 (Yellow Spring Inc., United States). Dissolved inorganic nutrients were measured in triplicate via the spectrophotometric method (Strickland and Parsons, 1972).

### Microscopic observation

Microalgae were analyzed and counted in triplicate under an inverted microscope (Nikon, TE2000-U, Japan) with 0.1 mL of the fixed samples at 400–600 times magnification. Morphological identification is performed at the lowest possible taxonomic level based on the taxonomic literatures (Tomas et al., 1997; Hallegraeff et al., 2004; Omura et al., 2012; Lassus et al., 2016).

### DNA extraction, PCR amplification, and sequencing

Environmental DNA (eDNA) is extracted and purified from filtered samples using the HiPure Plant DNA Mini Kit (Majorbio Bio-Pharm Technology Co. Ltd., China) according to the manufacturer’s protocol. The DNA concentration and quality were assessed using a NanoDrop 2000 spectrophotometer (Thermo Scientific, United States) with OD260/280 ≈ 1.8 and concentration > 10 ng/μL. The extracted eDNA samples were stored at −80°C for PCR amplification and sequencing.

Primer 3NDf 5′-GGCAAGTCTGGTGCCAG-3′ (Cavalier-Smith et al., 2009) and V4_euk_R2 5′-ACGGTATCTATCTCTTCG-3′ (Bråte et al., 2010) were used to amplify the V4 region of 18S rRNA gene by an ABI GeneAmp® 9700 PCR thermocycler (ABI, CA, United States). PCR system included 4 uL 5 × FastPfu Buffer, 2 uL 2.5 mM dNTPs, 0.8 uL forward primer (5 μM), 0.8 uL reverse primer (5 μM), 0.4 uL FastPfu polymerase, 0.2 uL bovine Serum albumin (BSA), 10 ng DNA template, and ddH_2_O to a final volume of 20 μL. The PCR cycle conditions were as following: (1) pre-denatured at 95°C for 3 min; (2) denatured at 95°C for 30 s, annealed at 55°C for 30 s, extended at 72°C for 45 s, 30 cycles; (3) extended at 72°C for 10 min and end at 4°C. The PCR products were run on 2% agarose gel, purified using the AxyPrep DNA gel extraction kit (Axygen Biosciences, Union City, CA, United States) according to the manufacturer’s instructions, and quantified using the Quantus™ fluorometer (Promega, United States). The purified amplifiers were collected in equimolar quantities. Sequencing was performed on the Illumina MiSeq PE300 platform (San Diego, United States) according to the standard protocol of Majorbio Bio-Pharm Technology Co. Ltd. (Shanghai, China). Raw data was deposited to the National Center for Biotechnology Information (NCBI) Sequence Read Archive (SRA)^1^ with the accession number PRJNA1069115.

### Data processing

Raw FASTQ files were de-multiplexed using an in-house perl script, and then quality-filtered by fastp (version 0.23.4, https://github.com/OpenGene/fastp) and merged by FLASH (version 1.2.11, http://www.cbcb.umd.edu/software/flash). Only overlapping sequences > 10 bp were assembled with the maximum mismatch ratio of 0.2. The optimized sequences were clustered into operational taxonomic units (OTUs) using USEARCH (version 11.0.667)-UPARSE (Edgar, 2013) with 97% sequence similarity level. The most abundant sequence for each OTU was selected as a representative sequence. Probable chimeric sequences were identified and removed using the UCHIME2 software package (Edgar, 2016). Chloroplast and mitochondria DNA reads were removed via QIIME2 (version 2023.5, https://qiime2.org/). The classification of each OTU was annotated by RDP Classifier (version 2.14) using a confidence threshold of 0.7 against the SILVA 18S rRNA gene database (version 138.1, https://www.arb-silva.de/). Algal OTUs were confirmed and selected by the taxonomic information in AlgaeBase (Guiry and Guiry, 2024, https://www.algaebase.org/), and the algal sequences were used for further analysis.

Those algal OTUs, unannotated against SILVA, were further searched against the NCBI database (https://www.ncbi.nlm.nih.gov/, searched in September 2023) using BLAST with a percent identity cutoff 97% and an e-value cutoff close to zero (≤10^−30^). The classification of algae was according to the up-to-date system suggested by Guiry (2024) and the AlgaeBase (Guiry and Guiry, 2024). HAB and/or bloom species were confirmed in the online version of the IOC-UNESCO Taxonomic Reference List of Harmful Micro Algae (Lundholm et al., 2009) or had been reported as a HAB or bloom species in previous studies.

In order to understand the bias between our primer and the classic V4 primer from Stoeck et al. (2010), and also the influence of different similarity and database on the analysis results, we have compared the two pairs of primers by metabarcoding four samples (S2, S4, S6, and S9) collected from this study using OTU assignment with 97% similarity against SILVA database and ASV assignment with 100% similarity against PR2 database. The results are listed in Supplementary Table S1. The results showed that the primer 3NDf and V4_euk_R2 increased the diversity and relative abundance of algal sequences, especially for those of dinoflagellates. While the classic V4 primers from Stoeck et al. (2010) obtained more relative abundance of diatoms. The results were comparable using SILVA database and PR2 database for the same primer, and also OTU assignment with 97% similarity and ASV assignment with 100% similarity. As the purpose of our study is to understand the diversity and distribution of HAB species, e.g., dinoflagellates, so we chose the primer 3NDf and V4_euk_R2 (Cavalier-Smith et al., 2009; Bråte et al., 2010).

### Statistical analyses

DNA reads in each sample were normalized to the fewest reads in this study (88,072 reads). Alpha diversity indices of microalgal community, including richness, Shannon, Simpson, and Pielou indices were calculated. Venn diagrams were calculated and plotted based on different samples. Bray-Curtis (BC) index was measured using vegdist package, and non-metric multidimensional scaling (NMDS) was constructed based on BC index using vegan and ggplot2 packages. Permutation multivariate analysis of variance (PERMANOVA) was performed using the adonis function to test group differences. The bubble charts of dominant taxa were drawn with the R packages ggplot2, reshape2 and forcats. All these analyses were performed using the R4.1.0.

## Results

### Microalgal community revealed by metabarcoding

**Figure 2:**
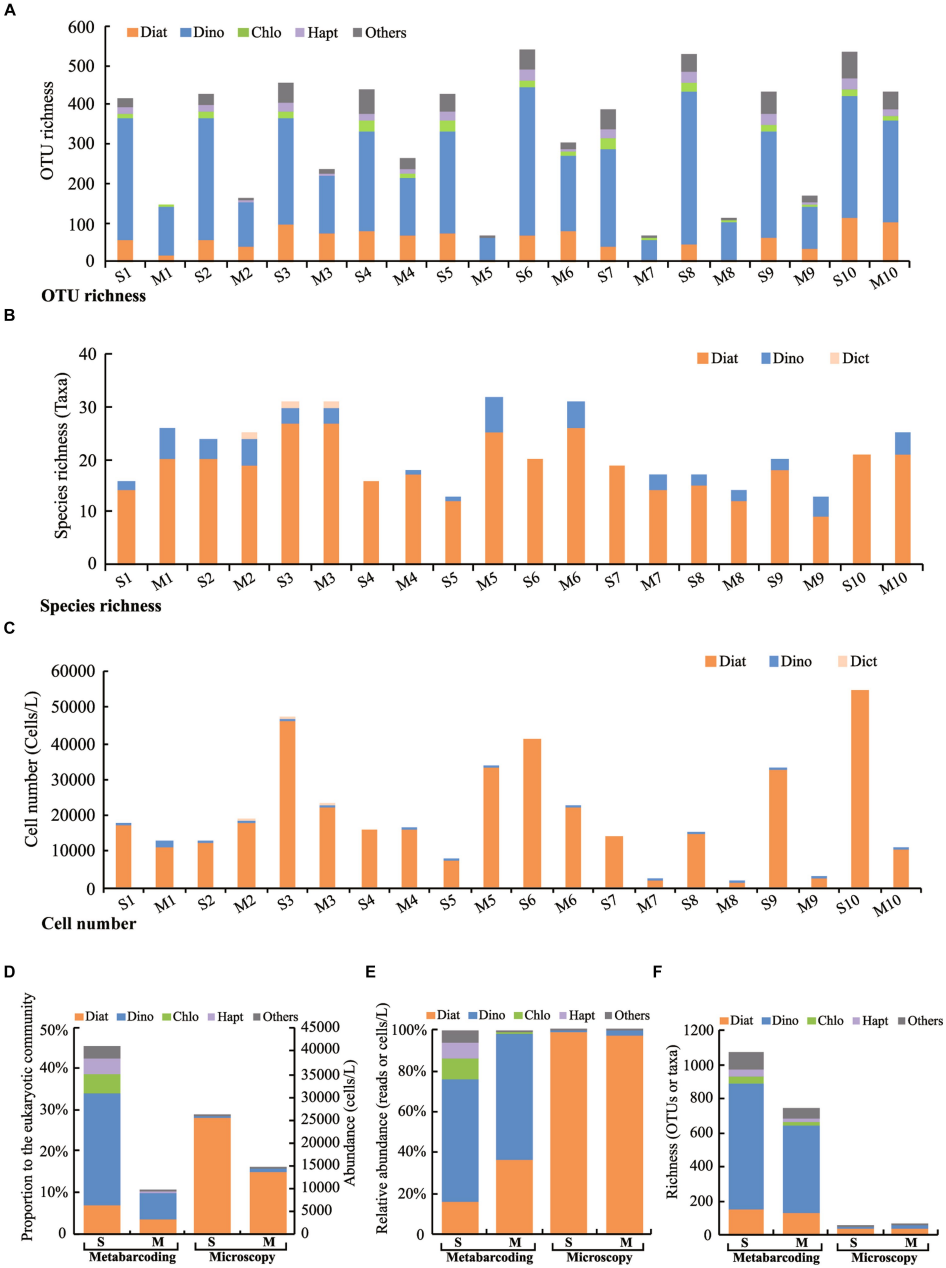
Microalgal community structure revealed by metabarcoding and microscopy. **(A)** OTU richness in each sample by metabarcoding, **(B)** Species richness in each sample by microscopy, **(C)** Cell number in each sample by microscopy, **(D)** Proportion of microalgal DNA reads to the eukaryotic community and cell number in the surface water samples (S) and micro-plankton samples (M), **(E)** Relative abundance of different groups of microalgae to the microalgal community, **(F)** Richness of different groups of microalgae in the surface water samples (S) and micro-plankton samples (M). S1–S10, Surface water samples; M1–M10, Micro-plankton samples; Diat, Diatoms including those in Bacillariophyceae, Mediophyceae, and Coscinodiscophyceae; Dino, Dinophyta; Chlo, Chlorophyta; Hapt, Haptophyta; Dict, Dictyochophyceae; Others, All other microalgal groups.

Altogether 1,167 eukaryotic algal OTUs were recorded, including 151 OTUs of diatoms (those in classes of Bacillariophyceae, Mediophyceae, and Coscinodiscophyceae), 831 OTUs in Dinophyta (dinoflagellates), 48 OTUs in Chlorophyta, and 35 OTUs in Haptophyta. The microalgal OTU richness ranged from 65 to 539 OTUs, and was generally lower in the micro-plankton samples than in the surface water samples (Figure 2A).

The eukaryotic algae detected in this study included 144 genera in 23 classes of 8 phyla (Supplementary Table S2). There were 308 OTUs (26.39%) identified at the species level, and 632 OTUs (54.16%) at the genus level. Altogether 202 species were detected in this study, including 74 species of diatoms, 65 species of dinoflagellates, 27 species of chlorophytes, 7 species of haptophytes, 15 species of cryptophyceans, 5 species of chrysophyceans, 4 species of raphidophyceans, and 5 species in other five classes (Supplementary Table S3). There were 198 and 166 species recorded in surface water (S) and micro-plankton (M) samples, respectively.

Dinoflagellates dominated in both surface water and micro-plankton samples, which accounted for about 60% of the algal DNA reads (Figure 2E). The microalgal community structure differed between the surface water and micro-plankton samples, reflected by lower relative DNA reads to the eukaryotic community, lower cell number (Figure 2D), and lower OTU richness (Figure 2F) in micro-plankton samples compared to those in surface water samples. Although contribution of diatom reads to the microeukaryotic community was much higher in surface water samples (7.48% on average) than that in micro-plankton samples (3.89% on average; Figure 2D), the percentage of diatom reads to microalgal reads in surface water samples (16.09%) was lower than that in micro-plankton samples (36.30%; Figure 2E). In addition, the relative reads of the nano-sized microalgae, such as chlorophytes and haptophytes, were significantly higher in surface water samples (8.01%–10.06%) than those in micro-plankton samples (mostly <1%) (Figure 2E). There were 1,068 and 747 OTUs of the eukaryotic microalgae detected in the surface water and micro-plankton samples, respectively (Figure 2F). The species compositions were similar in surface water and micro-plankton samples, and dinoflagellates were most diverse group, followed by diatoms, chlorophytes, and haptophytes (Figure 2F).

### Morphological identification

A total of 62 taxa (40 taxa at the species level) were identified in 40 genera of six classes in two microalgal phyla in this study, including 43 taxa of diatoms, 18 taxa of dinoflagellates, one taxon in Dictyochophyceae (Table 1). The number of microalgal species observed in each sample ranged from 13 to 32 taxa (Figure 2B), with an average of 21 taxa. Species richness was slightly higher in the micro-plankton samples (53 taxa) than in the surface water samples (44 taxa) (Figure 2F). Species richness of diatoms was the highest in all the samples, ranging from 9 to 27 taxa per sample (Figure 2B). Microalgal cell number ranged from 1.55 × 10^3^ cells/L to 5.47 × 10^4^ cells/L per sample (Figure 2C). On the whole, microalgal cell numbers were significantly higher in surface water samples than in microplankton samples (*p* < 0.01) (Figure 2C), with averages of 2.59 × 10^4^ and 1.43 × 10^4^ cells/L in surface water and micro-plankton samples, respectively (Figure 2D). Diatoms dominated in microalgal community with the relative abundances of 85.37%–100% and an average of 96.65% (Figure 2E).

**Table 1:** Comparison of microalgal community structure between light microscopy (LM) and metabarcoding (MB) analyses.

	Relative abundance (%)		Species richness		Species identified (taxa)		Genera identified (taxa)		Shared taxa		Unique genera		Unique species		HAB species			
	LM	MB	LM (taxa)	MB (OTU)	LM	MB	LM	MB	Shared species	Shared genera	LM	MB	LM	MB	LM		MB	
															S	M	S	M
Diat	98.25	12.94	43	151	26	74	28	39	9	20	8	19	17	65	8	11	16	16
Dino	1.65	71.21	18	831	13	65	11	53	5	9	2	44	7	60	5	7	12	12
Chlo		4.11		48		27		22				22		27				
Hapt		3.00		35		7		8				8		7			3	2
Others	0.1	8.76	1	102	1	29	1	22	0	0	1	22	1	29	0	0	3	3
Overall	100	100	62	1,167	40	202	40	144	14	29	11	115	25	188	13	18	34	33

LM, Light microscopy; MB, Metabarcoding; Diat, Diatoms; Dino, Dinoflagellates; Chlo, Chlorophyta; Hapt, Haptophyta; Others, All other microalgal groups; S, Surface water samples; M, Micro-plankton samples.

### Venn and non-metric multidimensional scaling analyses

**Figure 3:**
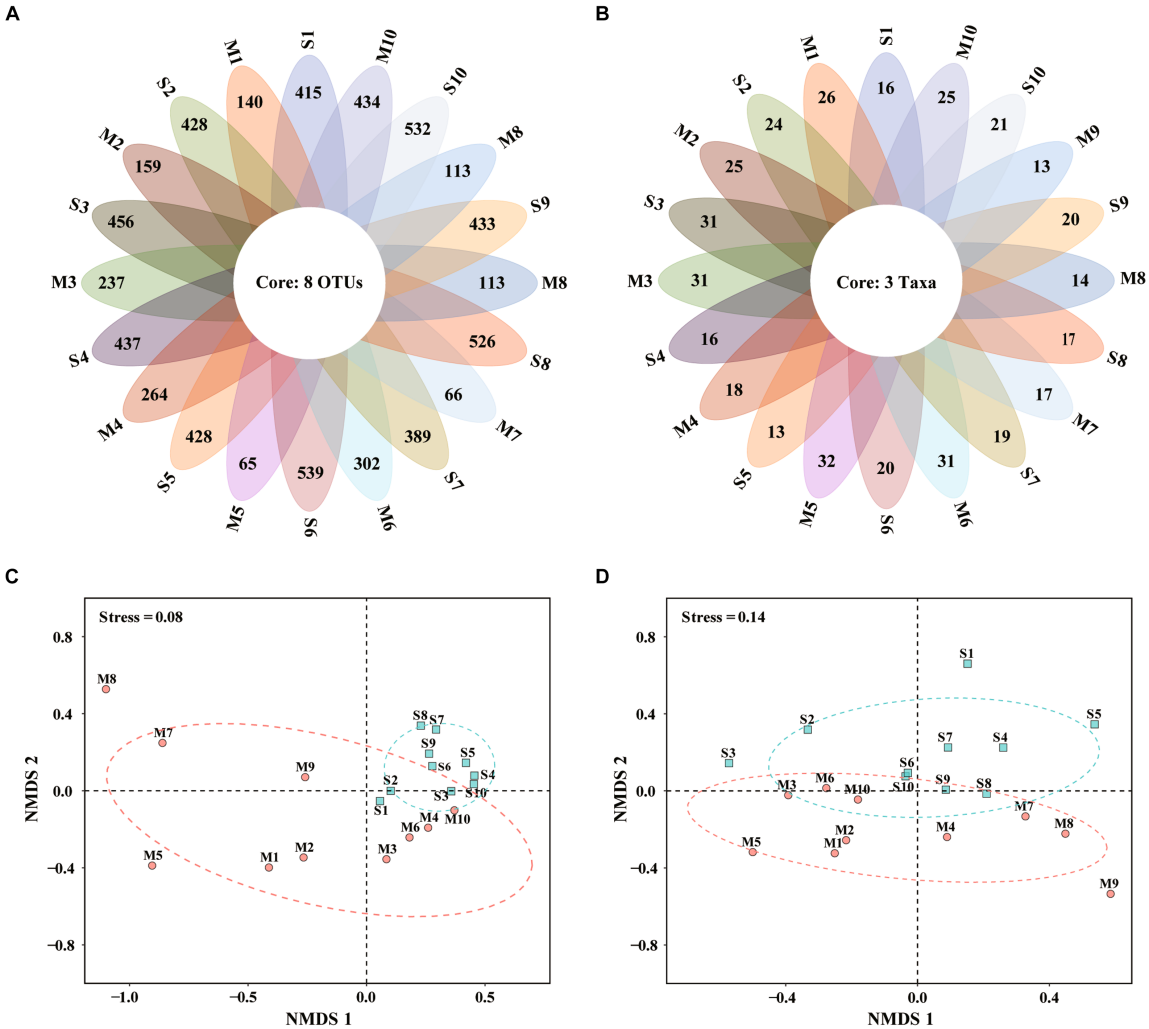
Venn diagrams and non-metric multidimensional scaling (NMDS) based on eukaryotic microalgal community revealed by metabarcoding **(A,C)** and microscopy **(B,D)**. S1–S10, Surface water samples; M1–M10, Micro-plankton samples.

The Venn diagram highlighted the differences of algal communities among the 20 samples (Figures 3A,B). There were only 8 OTUs and 3 taxa shared among all samples by metabarcoding and morphological observation, respectively. The results suggested different microalgal species composition among samples.

Non-metric multidimensional scaling (NMDS) well differentiated surface water and micro-plankton samples analyzed by metabarcoding (Figure 3C) and microscopic observation (Figure 3D). Based on metabarcoding data (Figure 3C), 10 surface water samples clustered together in the positive axis of NMDS1, indicating similar community structure among them. While micro-plankton samples scatteredly distributed at the second, third and fourth quadrants (Figure 3C), suggesting more diverse communities. Based on microscopic data (Figure 3D), surface water samples were mostly located in the first and second quadrants, while micro-plankton samples distributed in the third and fourth quadrants. There were significant differences between surface water samples and micro-plankton samples based on data revealed by both analyses (*R*^2^ = 0.321, *p* < 0.001 for metabarcoding, *R*^2^ = 0.123, *p* < 0.05 for microscopy, PERMANOVA; Table 2). The results suggested that the differences of microalgal community structure between different cell size fractions (all sizes vs. 20–200 μm) were much greater than those among different stations.

**Table 2:** Permutational multivariate analysis of variance (PERMANOVA) to evaluate the distances between surface water samples (S) and micro-plankton samples (M) in the Non-metric multidimensional scaling (NMDS).

Method	Pairs	Df	Sums of Sqs	F. Model	*R* ^2^	p. value	p. adjusted
Metabarcoding	S vs. M	1	1.872	8.524	0.321	0.001	0.001^**^
Microscopy	S vs. M	1	0.531	2.530	0.123	0.012	0.012^*^

^*^p < 0.05, ^**^p < 0.01.

### Distribution of abundant genera

**Figure 4:**
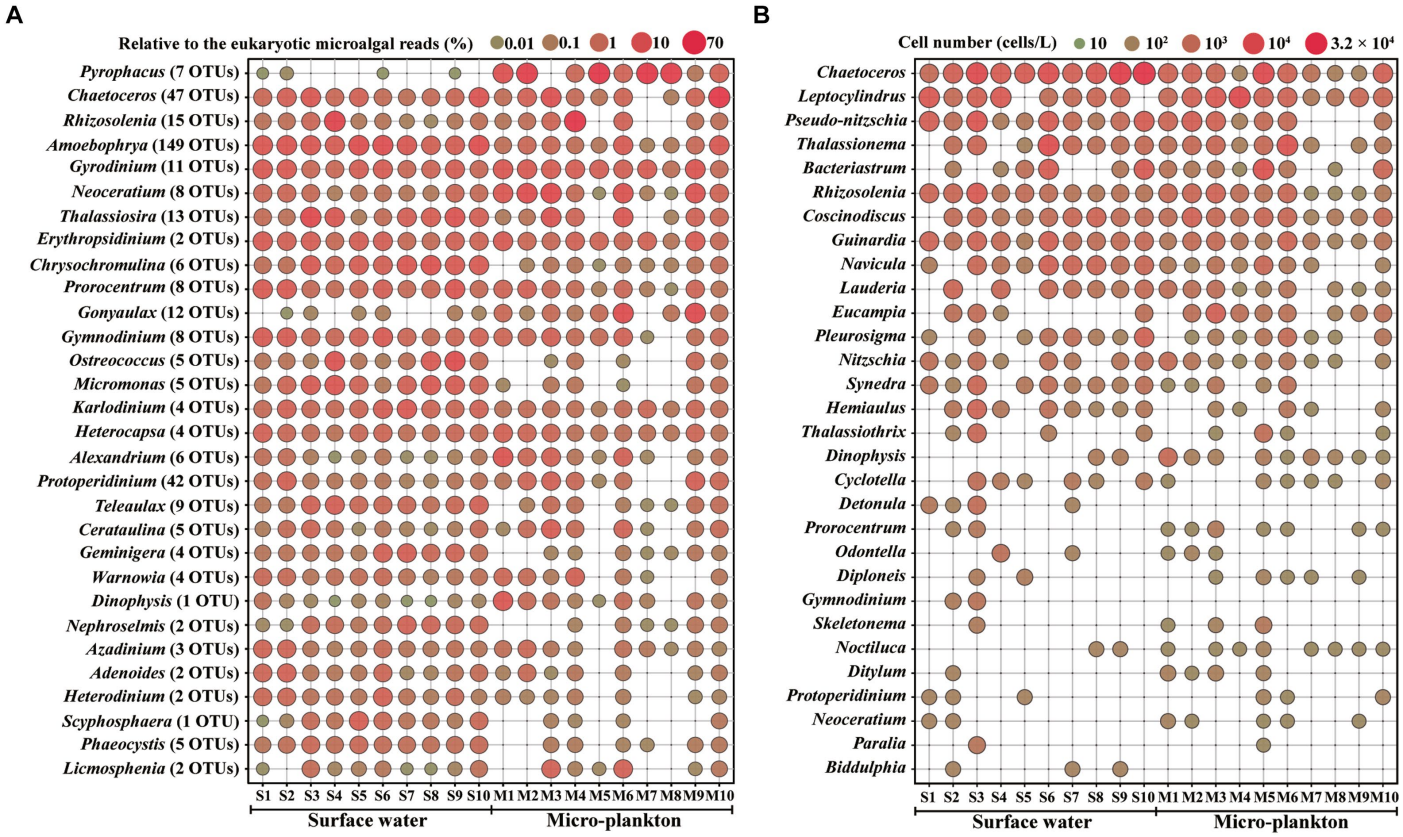
Distribution of the top 30 abundant genera by metabarcoding **(A)** and microscopy **(B)**. S1–S10, Surface water samples; M1–M10, Micro-plankton samples.

Figure 4 illustrates the distribution of the top 30 dominant genera based on metabarcoding (Figure 4A) and microscopic analyses (Figure 4B). *Pyrophacus* ranked the first abundant genus in metabarcoding dataset, and occurred abundantly in the micro-plankton samples (Figure 4A). The parasitic dinoflagellate *Amoebophrya* occurred abundantly in all samples, and ranked the top 4th genus. *Gyrodinium, Neoceratium*, *Prorocentrum*, *Gymnodinium*, *Karlodinium*, *Heterocapsa* were the common dominant dinoflagellate genera, and they were present in all samples. Other dominant dinoflagellate genera, such as *Gonyaulax*, *Alexandrium*, *Protoperidinium*, and *Dinophysis* were found in most samples. *Chaetoceros*, *Rhizosolenia*, and *Thalassiosira* were the most dominant diatoms, which ranked the top 2nd, 3rd, and 7th abundant genera, respectively. Most of the dominant taxa occurred more widely in surface water samples, however some micro-sized dinoflagellates and colony diatoms were more abundant in the micro-plankton samples, for example the dinoflagellates *Pyrophacus*, *Gyrodinium*, and *Neoceratium*, and the diatoms *Chaetoceros*, and *Rhizosolenia*. On the other hand, the nano-sized microalgae such as the haptophytes *Chrysochromulina*, *Scyphosphaera*, and *Phaeocystis*, the chlorophytes *Ostreococcus*, *Micromonas*, and *Nephroselmis*, the cryptophyceans *Teleaulax* and *Geminigera*, occurred abundantly in surface water samples (Figure 4A).

More than one OTUs were assigned to each species, for example 9 OTUs in *Rhizosolenia setigera*, 7 OTUs in *Pyrophacus steinii*, 6 OTUs in *Teleaulax amphioxeia*, 5 OTUs in *Gonyaulax spinifera*, and 5 OTUs in *Protoperidinium conicum* (Supplementary Table S3). Among the dominant genera, 149 OTUs were attributed to *Amoebophrya*, 47 OTUs to *Chaetoceros*, and 42 OTUs to *Protoperidinium* (Figure 4A).

The top 30 dominant genera based on microscopic analysis included 25 genera of diatoms and 5 genera of dinoflagellates (Figure 4B). Diatoms occupied all of the top 16 dominant genera. Most of these diatom taxa either belong to the micro-sized diatoms with cell size > 20 μm or to the colony diatoms, which are easily to be observed by microscopy. Only three genera, *Chaetoceros*, *Rhizosolenia*, and *Guinardia*, were present in all samples. Dinoflagellate genera such as *Dinophysis*, *Prorocentrum*, *Noctiluca*, and *Neoceratium*, occurred more widely and abundantly in the micro-plankton samples. The dominant genera identified by both microscopy and metabarcoding were *Chaetoceros*, *Rhizosolenia*, *Dinophysis*, *Prorocentrum*, *Gymnodinium, Protoperidinium*, and *Neoceratium*.

### Metabarcoding revealed high diversity of microalgal and HAB species

**Figure 5:**
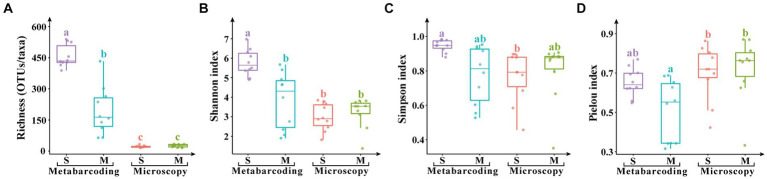
Alpha diversity indices of eukaryotic microalgae in the surface water samples (S) and the micro-plankton samples (M). **(A)** Species/ASV richness, **(B)** Shannon index, **(C)** Simpson index, **(D)** Pielou index. The significant difference of alpha indices among samples and analytical methods was performed by TukeyHSD test in R4.0.1. Data bars carrying different letter designations **(A–C)** indicated significant difference (*p* < 0.05), and the same and similar letter designations (e.g., a and ab, b and ab) denoted no significant difference (*p* > 0.05).

Metabarcoding revealed significantly higher microalgal OTU/species richness and Shannon index (*p* < 0.05), and richness and Shannon index were significantly higher in surface water samples than the micro-plankton samples (*p* < 0.05; Figure 5). Simpson index was comparable among samples and between the two analysis methods, however Pielou index was significantly higher revealed by microscopic observation than by metabarcoding.

The microalgal community showed significant differences between the results from microscopic observation and metabarcoding analysis. Diatoms predominated in the microalgal community based on microscopic observation, and dinoflagellates dominated based on metabarcoding analysis (Table 1). Metabarcoding annotated more species (202 species) than morphospecies (40 species) observed by microscopy. On the other hand, Chlorophyta, Haptophyta, Chrysophyceae, Cryptophyceae, Chlorarachniophyceae, Prasinodermatophyceae, and Rhodophyceae were detected in metabarcoding but overlooked in microscopy (Supplementary Table S2) due to their cryptic characteristics, such as small size and fragile cell walls. There were 14 species and 29 genera shared between the two methods (Table 1). Twenty-five and 188 unique species, and 11 and 115 unique genera were recorded by microscopy and metabarcoding, respectively (Table 1).

Altogether 48 algal bloom and/or HAB species were detected in this study revealed by both methods, including 24 diatoms, 18 dinoflagellates, and each of three species in Haptophyta and Raphidophyceae (Table 3). Metabarcoding detected much more bloom and/or HAB species (34 species) than those by microscopy (19 species). Actually, some HAB taxa were only detected to the genus level rather than the species level by microscopy, which cannot be sure whether to be HAB species. More HAB species were detected in the micro-plankton samples by microscopy, however they were comparable in the surface water and micro-plankton samples detected by metabarcoding (Table 1).

**Table 3:** List of algal bloom and/or harmful algal bloom (HAB) species detected in this study by light microscopy (LM) and metabarcoding (MB).

Phylum	Class	Species	Types of HAB	LM	MB
Heterokontophyta	Bacillariophyceae	*Bacillaria paxillifera*	Bloom (Jiang et al., 2022)	+	
		*Nitzschia longissima*	Bloom (Fiala et al., 2006)	+	
		*Nitzschia navis-varingica*	HAB, DA (Lundholm et al., 2009)		+
		*Pseudo-nitzschia caciantha*	HAB, DA (Lundholm et al., 2009)		+
		*Thalassionema frauenfeldii*	Bloom (Milham-Scott, 2021)	+	
		*Thalassionema nitzschioides*	Bloom (Makhlough et al., 2019)	+	
	Coscinodiscophyceae	*Guinardia flaccida*	Bloom (Hallegraeff et al., 2004)	+	+
		*Paralia sulcata*	Bloom (McQuoid and Nordberg, 2003)	+	
		*Rhizosolenia styliformis*	Bloom (Kemp et al., 2000)	+	
	Mediophyceae	*Chaetoceros curvisetus*	Bloom (Hallegraeff et al., 2004)		+
		*Chaetoceros decipiens*	Bloom (Hallegraeff et al., 2004)		+
		*Chaetoceros diadema*	Bloom (Gogorev and Samsonov, 2016)		+
		*Chaetoceros pseudo-curvisetus* ^†^	Bloom (Hallegraeff et al., 2004)		+
		*Chaetoceros socialis* ^†^	Bloom (Hallegraeff et al., 2004)		+
		*Ditylum brightwellii*	Bloom (Rynearson and Armbrust, 2000)	+	
		*Lauderia annulata*	Bloom (Hallegraeff et al., 2004)	+	+
		*Leptocylindrus danicus* ^†^	Bloom (Hallegraeff et al., 2004)	+	+
		Skeletonema costatum*	Bloom (Hallegraeff et al., 2004)	+	
		*Skeletonema tropicum*	Bloom (Gu et al., 2012)		+
		*Thalassiosira curviseriata*	Bloom (Hallegraeff et al., 2004)		+
		*Thalassiosira diporocyclus*	Bloom (Hallegraeff et al., 2004)		+
		*Thalassiosira mala* ^†^	Bloom (Hallegraeff et al., 2004)		+
		*Thalassiosira minima*	Bloom (Guinder et al., 2012)		+
		*Thalassiosira gravida*	Bloom (Mikhail et al., 2020)		+
	Raphidophyceae	*Chattonella subsalsa*	HAB, Ichthyotoxin (Lundholm et al., 2009)		+
		*Fibrocapsa japonica* ^†^	HAB, Ichthyotoxin (Lundholm et al., 2009)		+
		*Heterosigma akashiwo*	HAB, Ichthyotoxin (Lundholm et al., 2009)		+
**Dinoflagellata**					
	Dinophyceae	*Alexandrium leei*	HAB, PSP (Lundholm et al., 2009)		+
		*Alexandrium minutum*	HAB, PSP (Lundholm et al., 2009)		+
		*Alexandrium fraterculus*	HAB (Hallegraeff et al., 2004)		+
		*Dinophysis caudata*	HAB, DSP (Lundholm et al., 2009)	+	+
		*Gambierdiscus toxicus*	HAB, CFP (Lundholm et al., 2009)	+	
		*Gonyaulax spinifera*	HAB, YTX (Lundholm et al., 2009)		+
		*Gymnodinium catenatum*	HAB, PSP (Lundholm et al., 2009)		+
		*Karlodinium veneficum*	HAB, Karlotoxins (Lundholm et al., 2009)		+
		*Lingulodinium polyedra*	HAB, YTX (Lundholm et al., 2009)		+
		*Margalefidinium fulvescens*	HAB, Ichthyotoxin (Lundholm et al., 2009)		+
		*Prorocentrum dentatum*	Bloom (Wang and Huang, 2003)	+	
		*Prorocentrum micans* ^†^	Bloom (Hallegraeff et al., 2004)	+	
		*Prorocentrum sigmoides*	Bloom (Hallegraeff et al., 2004)	+	
		*Protoceratium reticulatum*	HAB, YTX (Lundholm et al., 2009)		+
		*Scrippsiella acuminata* ^†^	Bloom (Wang et al., 2007)		+
		*Tripos furca*	HAB (Hallegraeff et al., 2004)	+	
		*Tripos muelleri*	HAB (Er et al., 2018)	+	
	Noctilucophyceae	*Noctiluca scintillans* ^*†^	Bloom (Hallegraeff et al., 2004)	+	+
**Haptophyta**					
	Coccolithophyceae	*Phaeocystis globosa* ^*†^	HAB, Hemolysin (Lundholm et al., 2009)		+
		*Phaeocystis pouchetii*	HAB, Ichthyotoxin (Lundholm et al., 2009)		+
		*Prymnesium parvum*	HAB, Ichthyotoxin (Lundholm et al., 2009)		+
	Total			19	34

^+^The occurrence of the species. ^*^Historical algal bloom species (Xu et al., 2019). ^†^Taxa shared with those reported in He et al., 2023. MB, Metabarcoding; LM, Light microscopy; CFP, ciguatera fish poisoning; DA, Domoic acid; DSP, Diarrhetic shellfish poisoning; PSP, Paralytic shellfish poisoning; YTX, Yessotoxin.

## Discussion

### Microalgal community structure in coastal sea areas of the Beibu Gulf

**Figure 6:**
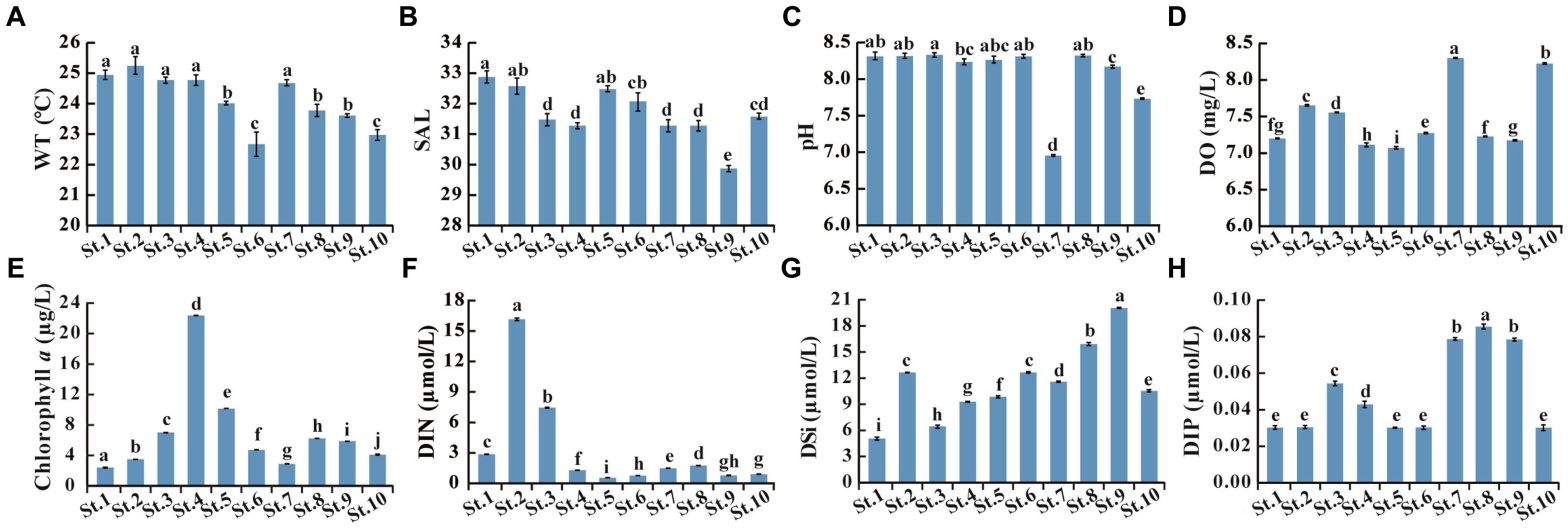
Environmental parameters measured in the 10 stations. **(A)** WT, Water temperature; **(B)** SAL, Salinity; **(C)** pH; **(D)** DO, Dissolved oxygen; **(E)** Chlorophyll a; **(F)** DIN, Dissolved inorganic nitrogen as the sum of NO_2_^−^-N, NO_3_^−^-N, and NH_4_^+^-N; **(G)** DSi, Dissolved inorganic silicate (SiO_3_^2−^-Si); **(H)** DIP, Dissolved inorganic phosphorus (PO_4_^3−^-P). Data bars carrying different letter designations (a–h) indicated significant difference (*p* < 0.05), and the same and similar letter designations (e.g., a and ab, b and ab) denoted no significant difference (*p* > 0.05).

Microalgal communities in the Beibu Gulf have been studied based on microscopic observation (Xu et al., 2014; Wang et al., 2015; Qin et al., 2023). However, the nano-sized microalgae are often overlooked, and similar and cryptic taxa cannot be accurately distinguished under light microscopic observations (Esenkulova et al., 2020). To our knowledge, this is the first attempt to study the microalgal community and HAB species in the Beibu Gulf combining microscopic observation and metabarcoding data. Microscopic observations showed that diatoms dominated the microalgal community, which agreed with those in previous studies based on microscopic observations (Sun et al., 2011; Xu et al., 2014; Wang et al., 2015). The low degree of overlap of microalgal taxa in Venn diagram (Figures 3A,B) suggested differences in microalgal community structure between samples, even if they were collected from close stations due to fluctuant environmental parameters among stations (Figure 6).

Metabarcoding revealed an increased abundance of dinoflagellates and a reduction in diatoms. The different results between metabarcoding and microscopy may be primarily due to substantial variation in 18S rRNA gene copy number among microalgal species (Zhu et al., 2005). In general, dinoflagellates have a higher 18S rRNA gene copy number than other microalgae (Gong and Marchetti, 2019), and a high logarithmic correlation between DNA copy number and cell size was observed (Zhu et al., 2005). Since nano-sized diatoms (<20 μm), such as *Chaetoceros* spp., *Leptocylindrus danicus*, and *Pseudo-nitzschia* spp., dominated in this study (Figure 4B), their gene copies are lower than those of dinoflagellate cells. High variation in the copy number of the 18S rRNA gene may lead to a rapid decline in consistency between the DNA reads and the actual organism abundances (Gong and Marchetti, 2019). The predominance and bias of dinoflagellates in microalgal metabarcoding have been widely reported (Xu et al., 2017; Song et al., 2019; Liu et al., 2020; Cui et al., 2021; Wang Z. H. et al., 2022).

Microalgal community structure differed significantly between the surface water and micro-plankton samples (Figures 3C,D), reflected by higher abundance (cell number and proportion within the eukaryotic community), richness (taxa and OTUs), and alpha diversity in the surface water samples (Figures 2, 5). We collected micro-plankton samples in order to better understand distribution of the micro-sized dinoflagellates especially those of HAB taxa. Dinoflagellates occurred more abundantly and widely in micro-plankton samples based on microscopic observations (Figures 2D,E, 4B). Meanwhile, more HAB taxa were recorded in micro-plankton samples (18 taxa) than those in the surface water samples (13 taxa) based on microscopic analysis (Table 1). However, HAB taxa detected by metabarcoding were comparable in the surface water and micro-plankton samples. Furthermore, a large number of nano- and pico-sized microalgae, such as those in chlorophytes, cryptophytes, and haptophytes, are lost in the micro-plankton samples even by metabarcoding. Our results suggest that HAB species may be more abundant in the micro-plankton samples based on microscopic observations, whereas it is not necessary to collect concentrated micro-plankton samples when studying HAB species by metabarcoding.

### Metabarcoding revealed high bio-diversity of microalgae and gaps between metabarcoding and microscopy

Twenty-three classes in eight microalgal phyla were detected by metabarcoding, however only six classes in two phyla were identified by microscopy (Supplementary Table S2). Numerous taxa (species and genera) in Chlorophyta, Haptophyta, Rhodophyta, Chlorarachniophyceae, Chrysophyceae, Cryptophyceae, and Prasinodermatophyceae detected in metabarcoding were not identified by microscopy (Supplementary Table S2). The results suggest metabarcoding revealing much higher bio-diversity than that by microscopy. Metabarcoding has a strong ability to detect the tiny, morphologically similar, fragile, and rare species (Salonen et al., 2019). Plenty of nano-sized chlorophytes, chlorarachniophyceans, chrysophyceans, haptophytes, and prasinodermatophyceans (<10 μm) were detected in this study (Supplementary Table S2). Lin et al. (2021) found abundant chlorophytes (mostly *Micromonas* and *Ostreococcus*) and chrysophyceans (*Paraphysomonas*) in 0.45–5-μm plankton community of the Beibu Gulf based on metabarcoding. However, majority of these tiny-sized species are missed in previous microscopic studies (Xu et al., 2014; Wang et al., 2015; Qin et al., 2023). In addition, metabarcoding distinguished morphologically similar or fragile genera to many species that cannot be identified to the species level under light microscopy, such as species in diatoms *Chaetoceros, Leptocylindrus*, and *Thalassiosira*, and dinoflagellates *Alexandrium, Azadinium*, and taxa in naked dinoflagellate order Gymnodiniales such as *Gymnodinium*, *Gyrodinium*, and *Karlodinium* etc. (Supplementary Table S2).

However, only 14 species and 29 genera were shared in both methods (Table 1), and even among the top 30 dominant genera, only 7 genera were detected in both microscopic and metabarcoding datasets (Figure 4). The results suggested a huge gap between the two methods. The reason for this phenomenon is firstly due to the low coverage at the species level for many taxa in the reference databases, which may hinder correct interpretation of metabarcoding data (Forster et al., 2016). Only 308 OTUs (26.39%) were identified at the species level, and 632 OTUs (54.16%) at the genus level after annotated against both the SILVA and NCBI databases in our study. Anyway, as new sequencing data are continuously contributing to the existing databases, the situation is likely to improve in the future. In addition, species identification based on traditional microscopic methods is restricted to those organisms with well-documented morphological characteristics and easy to be identified (Eland et al., 2012). Diatoms identified by microscopy in this study were mostly those with cell size > 20 μm or the colony taxa (Figure 4B; Supplementary Table S2). While morphological observation provides cell number of each taxon, and better reflects the real situation in the water column. The two databases can complement each other, and the combination of the two methods better reflect planktonic microalgal community in the water column.

### Occurrence of HAB species and potential risks of HABs

A total of 48 algal bloom and/or HAB taxa were identified in this study, however, only three species of them (*Skeletonema costatum*, *Noctiluca scintillans*, and *Phaeocystis globosa*) formed blooms previously in the Beibu Gulf (Xu et al., 2019). He et al. (2023) detected 37 HAB species by metabarcoding in one station during the eight surveys between 2016 and 2017, but only nine HABs were the same as in our study (Table 3). These results suggest rich and diverse HAB species in the Beibu Gulf, which may fluctuate greatly with sampling time.

Diatoms are the foundation of the global marine ecosystem, accounting for about 40% of the ocean’s total primary production and 20% of the Earth’s total primary production (Tréguer et al., 2018; Karlusich et al., 2020). Most diatoms do not produce toxins harmful to marine organisms. Sometimes they form algal blooms that result in basically harmless water discolorations; however, under special conditions, dense blooms may cause indiscriminate kills of fish and invertebrates through oxygen depletion (Hallegraeff et al., 2004). Two potential domoic acid (DA) producers, *Nitzschia navis-*var*ingica* and *Pseudo-nitzschia caciantha*, were detected in our study (Table 3). *Pseudonitzschia* spp. are the major DA producers, with 73 species recorded so far (Guiry and Guiry, 2024), of which 33 species/variants produce DA (Lundholm et al., 2009). DA has been detected in shellfish samples collected from the Beibu Gulf, with the maximum level of 401 μg/kg and the detection rate of 17.7% (Ji et al., 2022). *Pseudo-nitzschia* was the third top genus observed by microscopy (Figure 4B) with the average cell numbers of 1.7 × 10^3^ cells/L in our study. Based on metabarcoding, its relative DNA reads was outside the top 30 genera. Both abundances of *Pseudo-nitzschia* detected in this study and DA concentrations and detection rate reported by Ji et al. (2022) in the Beibu Gulf were much lower than those reported in the other sea areas of the southern Chinese coast (Wang Z. H. et al., 2022), suggesting relatively a low risk of DA poisoning events in this region.

Dinoflagellates are particularly important for HABs, because about 75–80% of known toxic microalgae belong to dinoflagellates (Cembella, 2003). A total of 18 harmful dinoflagellate taxa were identified in this study, and some were only found in microscopic analysis, such as *Gambierdiscus toxicus*, *Tripos furca*, and *T. muelleri* (Table 3). The results suggest that there are certain biases in the metabarcoding process and/or gaps in the sequence database for above genera. However, more diverse species in *Alexandrium* were found in metabarcoding, and more potentially harmful dinoflagellate species were detected (*Gonyaulax spinifera*, *Gymnodinium catenatum*, *Karlodinium veneficum*, *Lingulodinium polyedra*, *Margalefidinium fulvescens*, and *Protoceratium reticulatum*). Some of these HAB taxa have the potential to produce toxins that can cause toxic effects on human health through the food chain, for example, the paralytic shellfish poisoning (PSP) producers *Alexandrium* spp., diarrhetic shellfish poisoning (DSP) producer *Dinophysis caudata*, ciguatera fish poisoning (CFP) producer *Gambierdiscus toxicus*, yessotoxin (YTX) producers *Gonyaulax spinifera, Lingulodinium polyedra*, and *Protoceratium reticulatum*, and karlotoxins producers *Karlodinium veneficum* (Table 3). In addition, a large number of HAB species, which are potentially toxic to fish and shellfish and cause mass mortalities of aquaculture animals, were detected in the Beibu Gulf in our study, such as the dinoflagellate *Margalefidinium fulvescens*, the haptophytes *Phaeocystis globosa*, *Phaeocystis pouchetii*, and *Prymnesium parvum*, and the raphidophyceans *Fibrocapsa japonica*, *Heterosigma akashiwo*, and *Chattonella subsalsa* (Table 3). Our result suggested a high potential risk of HABs in the Beibu Gulf.

*Phaeocystis globosa* and *Noctiluca scintillans* are the main bloom-causative species in the Beibu Gulf, and their blooms have occurred frequently in the recent decades (Xu et al., 2019). A massive bloom caused by *Noctiluca scintillans* with a total coverage area of >20,000 km^2^ occurred in Qinzhou Bay of the Beibu Gulf in February 2021 (Xie et al., 2023). However, abundances of these two species were low revealed by both microscopic and metabarcoding analyses in this study. *Phaeocystis globosa* is a nano-sized haptophyte, which can form giant colonies during the bloom, and exists as solitary cells in the sea water during the rest of the time (Liang et al., 2023). The tiny solitary cells (3.5–4.5 μm) are easily overlooked under routine light microscopic observation, which makes them hard to be detected except during its blooms. *Noctiluca scintillans* is a heterotrophic dinoflagellate, which needs to feed on other small microalgae, and its outbreak usually follows the diatom blooms (Kopuz et al., 2014; Kitatsuji et al., 2019). Therefore, the low abundances of these two bloom species in our study were possibly because our sampling missed their growth seasons.

## Conclusion

In conclusion, metabarcoding revealed much higher microalgal diversity and species richness than those by morphological observation. Microalgal community in surface water samples demonstrated significantly higher OTU/species richness, alpha diversity, and abundance than those in micro-plankton samples. Metabarcoding and microscopy demonstrated different microalgal community, which was dominated by dinoflagellates and diatoms, respectively. Metabarcoding has a strong ability to detect the small-sized, morphologically similar, fragile and rare species. While morphological observation provides cell number of each taxon, and better reflects the real situation in the water column. The combination of morphological and metabarcoding approaches comprehensively reveals the microalgal community structure and diversity of HAB species.

## Data availability statement

The datasets presented in this study can be found in online repositories. The names of the repository/repositories and accession number(s) can be found in the article/Supplementary material.

## Author contributions

CW: Formal analysis, Writing – original draft. JG: Data curation, Methodology, Writing – review & editing. WL: Data curation, Investigation, Writing – original draft. JW: Methodology, Writing – review & editing. ZW: Conceptualization, Funding acquisition, Writing – original draft. QL: Conceptualization, Data curation, Writing – review & editing.

## Footnotes

^1^ https://www.ncbi.nlm.nih.gov/sra/
